# Title, abstract and keywords: a practical guide to maximize the visibility and impact of academic papers

**DOI:** 10.1098/rspb.2024.1222

**Published:** 2024-07-31

**Authors:** Patrice Pottier, Malgorzata Lagisz, Samantha Burke, Szymon M. Drobniak, Philip A. Downing, Erin L. Macartney, April Robin Martinig, Ayumi Mizuno, Kyle Morrison, Pietro Pollo, Lorenzo Ricolfi, Jesse Tam, Coralie Williams, Yefeng Yang, Shinichi Nakagawa

**Affiliations:** ^1^ Evolution & Ecology Research Centre, School of Biological, Earth and Environmental Sciences, The University of New South Wales, Sydney, New South Wales, Australia; ^2^ Division of Ecology and Evolution, Research School of Biology, The Australian National University, Canberra, Australian Capital Territory, Australia; ^3^ Theoretical Sciences Visiting Program, Okinawa Institute of Science and Technology Graduate University, Onna 904-0495, Japan; ^4^ Institute of Environmental Sciences, Jagiellonian University, Kraków, Poland; ^5^ Ecology and Genetics Research Unit, University of Oulu, Oulu, Finland; ^6^ School of Life and Environmental Sciences, Charles Perkins Centre, The University of Sydney, Sydney, New South Wales, Australia; ^7^ Department of Biology, Faculty of Science, Hokkaido University, Sapporo 060-0810, Japan; ^8^ Centre for Ecosystem Science, School of Biology, Earth and Environmental Sciences, The University of New South Wales, Sydney, New South Wales, Australia

**Keywords:** search engine optimization, academic publishing, online database search, journal policies, literature review, open access

## Abstract

In a growing digital landscape, enhancing the discoverability and resonance of scientific articles is essential. Here, we offer 10 recommendations to amplify the discoverability of studies in search engines and databases. Particularly, we argue that the strategic use and placement of key terms in the title, abstract and keyword sections can boost indexing and appeal. By surveying 230 journals in ecology and evolutionary biology, we found that current author guidelines may unintentionally limit article findability. Our survey of 5323 studies revealed that authors frequently exhaust abstract word limits—particularly those capped under 250 words. This suggests that current guidelines may be overly restrictive and not optimized to increase the dissemination and discoverability of digital publications. Additionally, 92% of studies used redundant keywords in the title or abstract, undermining optimal indexing in databases. We encourage adopting structured abstracts to maximize the incorporation of key terms in titles, abstracts and keywords. In addition, we encourage the relaxation of abstract and keyword limitations in journals with strict guidelines, and the inclusion of multilingual abstracts to broaden global accessibility. These recommendations to editors are designed to improve article engagement and facilitate evidence synthesis, thereby aligning scientific publishing with the modern needs of academic research.

## 1. Introduction

Scientific articles serve as the primary method for disseminating research findings. Between 1980 and 2012, global scientific output was estimated to increase by 8–9% every year, implying a doubling of scientific evidence approximately every 9 years [1]. Amid this burgeoning landscape, standing out becomes a research agenda in its own right. Ensuring that articles are well written and indexed in databases such as Scopus or Web of Science is akin to laying the first bricks in the foundation—it is important for discoverability, but not sufficient. Many articles, despite being indexed, remain undiscovered (coined the ‘discoverability crisis’ [2]). We argue that carefully crafting titles, abstracts and keywords is a critical step to increase the visibility and impact of scientific research.

Titles, abstracts and keywords are the primary marketing components of any scientific paper, and carefully designing these elements is crucial [3,4]. However, studies with appealing abstracts will not necessarily be discovered and cited because of a lack of search engine optimization [5]. Search engine optimization is the process of enhancing the findability of content by search engines. While often not discussed in the academic sphere, it is particularly relevant for scientific articles. To discover articles, academics often use a combination of key terms in scientific literature databases or search engines, and most databases leverage algorithms to scan the words in titles, abstracts and keywords to find matches. Failure to incorporate appropriate terminology could thus undermine readership. Other search engines such as Google Scholar may look through articles in their entirety [6]. Academics may also use other pathways to discover scientific articles, such as recommendations from colleagues or suggested content on social media. However, the same underlying principle remains—the absence of critical key terms means these articles would not surface in your search results, or those of your colleagues. Social media is also likely to recommend content that is most engaged by the user, and studies with inappropriate key terms may not appear as suggested content. Notably, keywords play an important role in the search ranking process. Choosing well-suited terms can often mean the difference between a study appearing at the top of the search results or being buried beneath a virtual pile of other documents. This is particularly important for databases that sort results by relevance, where the strategic use of keywords can significantly enhance an article’s visibility. Although the functioning of most relevance ranking algorithms is not publicly disclosed [7], it is reasonable to expect that articles containing search terms in the title or abstract will be ranked higher than other articles not containing these terms, or in more cryptic parts of the manuscript (e.g. in the methods). Additionally, not including relevant keywords impedes a study’s inclusion in literature reviews and meta-analyses, which often rely on database searches based on key terms in titles, abstracts and keywords [8,9].

Enhancing study discoverability is, however, ineffective if the abstract and title fail to engage the reader. Readers typically gauge the relevance of a study by briefly scanning the title and abstract. If these lack essential keywords or are mired in uncommon jargon, they may not capture the reader’s interest. An abstract that is well-structured, accurate, descriptive and written with a narrative can significantly influence whether a study is read thoroughly, sidelined onto a reading backlog, or ignored [3,4,10–12]. Therefore, the interplay between strategic keyword inclusion and compelling abstract and title composition serves as a bridge between discoverability and engagement, laying the groundwork for academic impact (i.e. whether the study is read, cited and/or used in future works). Although discoverability does not directly imply impact, papers with a larger readership tend to accumulate more citations [13–15] because we cannot cite what we do not discover.

**Figure 1.**
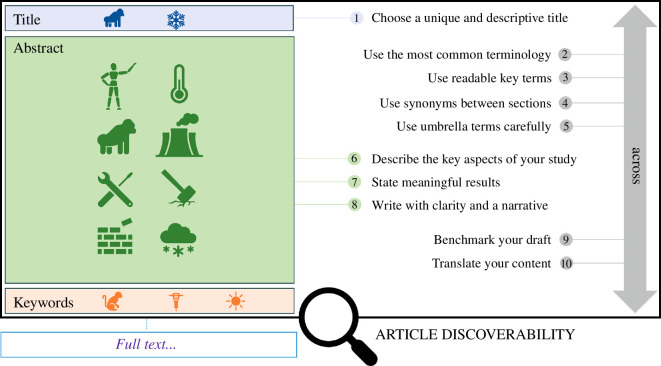
Ten strategic recommendations to improve the discoverability of scientific articles. Some recommendations are specific to a section, while others transcend multiple sections. The number refers to the sections of the guide (see main text).

**Figure 2.**
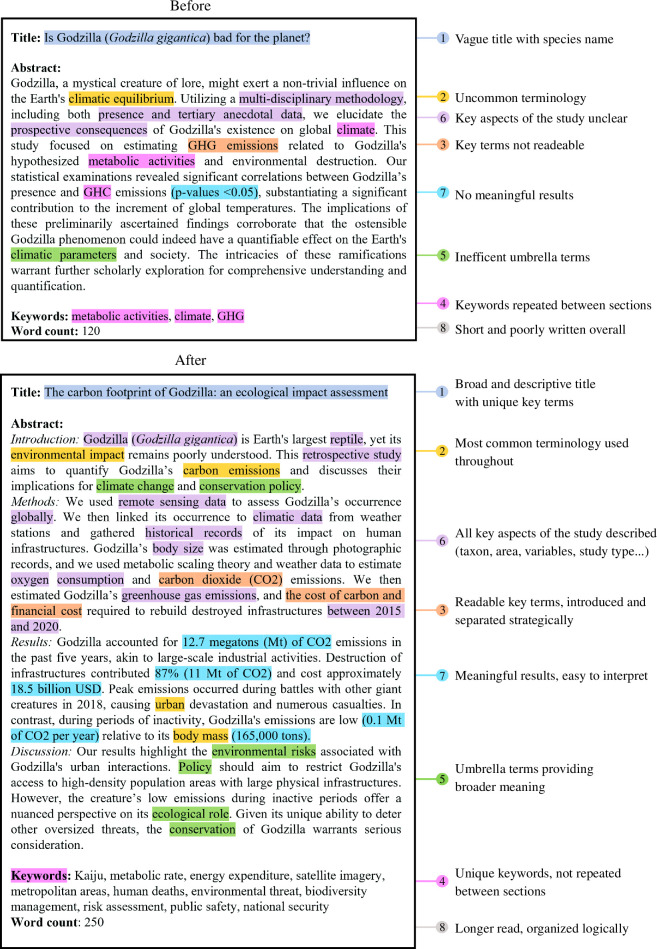
Example of strategic use of key terms. Above is a hypothetical abstract capped at 120 words that does not follow our recommendations. Below is a longer (250 words) abstract that follows our recommendations for crafting titles, abstracts and keywords. The text highlighted and numbers refer to specific parts of the guide (see main text). While the abstract is strictly structured following IMRAD in this example, the same abstract could be used without section headers for each section for journals not allowing structured abstracts.

Here, we propose recommendations to maximize the discoverability and impact of scientific articles. First, we offer a practical guide to crafting effective titles, abstracts and keywords for articles to augment their findability in search engines (figures 1 and 2), as well as additional considerations to maximize discoverability. These recommendations were generated through numerous workshops and discussions with the author team. Some recommendations are evidence-based, while others stem from our extensive experience in conducting systematic reviews and meta-analyses (mostly in ecology and evolutionary biology), which taught us lessons on maximizing discoverability and impact. Second, we surveyed 230 journals in the fields of ecology and evolutionary biology to evaluate how existing author guidelines may inadvertently hinder article discoverability. Indeed, some of our recommendations may conflict with existing author guidelines set by journals. Evaluating variations in the length and structure of abstracts, titles and keywords can thus highlight if editorial changes are needed to facilitate the adoption of our recommendations. Third, reflecting on our recommendations and literature survey, we suggest a set of recommendations for journal editors that aim to optimize the likelihood of published works being discovered and cited. Ultimately, these recommendations aim to enhance article engagement and facilitate evidence synthesis.

### (a) A practical guide to crafting titles, abstracts and keywords

#### (i) Choose a unique and descriptive title

Titles hold a pivotal role in scientific papers. From reviewers to readers, it is the first point of engagement [16]. It is thus not surprising that article discoverability and engagement can be shaped by the contents of their titles.

The relationship between title length and citation rates is a point of contention. While some studies suggest that shorter titles provide citation advantages [17–19], others find the opposite pattern [20,21] or no relationship [22–26]. Importantly, effects, when detected, are weak or moderate, suggesting that other features of articles may be more important than title length. In the field of ecology and evolutionary biology, titles have been getting longer without much consequence for citation rates [24,27]. However, exceptionally long titles (>20 words) tend to fare poorly during peer review [24]. For some search engines, lengthy titles may be trimmed owing to space limitations (e.g. when using a mobile device), which may impede discovery [5]. Therefore, avoiding excessively long titles is likely to be sensible.

The perceived scope of the title also seems to have an influence on a paper’s impact. A narrow-scoped title tends to have negative effects, with papers that include species names in titles receiving significantly fewer citations compared to papers that avoid this practice [24,25,27]. This suggests that framing your findings in a broader context can increase your study’s appeal to readers and editors. However, it is important not to inflate the scope of your study so that the title remains accurate, descriptive and informative [28,29] (figure 2). For instance, a study investigating the thermal tolerance of *Pogona vitticeps* could phrase its title as ‘thermal tolerance of a reptile’ rather than ‘thermal tolerance of reptiles’, the latter implying the study results are applicable to all reptiles.

Humour also appears to play a role in a paper’s future impact. While an earlier study [30] found this association varied across fields, this analysis did not correct for individual journal properties. Conversely, a recent study discovered that papers with titles that scored the highest for humour had nearly double the citation count as papers that received the lowest scores, even after accounting for self-citation rates [27]. Incorporating humour might be seen as risky, but this trend could signal a shift in perceptions, where a well-placed pun can enrich academic writing and engage the reader. Humorous titles are also more easily remembered, which may play a role in a study’s future impact [27]. However, authors should be mindful of accessibility when crafting titles to avoid alienating non-English speakers [31]. Funny titles often rely on cultural references that are far from universal, thus metaphors should be used with caution [32].

The art of crafting an engaging title is also complemented by a scientific consideration for accuracy and discoverability. Implementing stylistic structures such as incorporating humour is a progressive step, but attention must also be given to the integration of relevant key terms to accurately describe the content. For instance, humorous parts of the title may be used in conjunction with more descriptive information by separating the title with punctuation (e.g. colon). This way, titles can reach readers who enjoy humorous titles without compromising scientific integrity. A simple search of your title can also ensure the chosen title is distinct from other published articles, reducing the likelihood of your paper being overshadowed in the vast scientific literature.

#### (ii) Use the most common terminology

The terminology used in a scientific article is not merely descriptive. Key terms can be used strategically to enhance the discoverability of scientific research and their influence extends beyond the keyword section. The more we incorporate key terms or phrases that encapsulate the essence of our research, the more likely our work is to surface in broad database searches [5]. Emphasizing recognizable key terms, those frequently employed in the related literature, can significantly augment the findability of an article (figure 2). Papers whose abstracts contain more common and frequently used terms also tend to have increased citation rates [33,34]. Importantly, it is preferable to place the most common and important key terms at the beginning of the abstract, as not all search engines display the entire abstract [5]. It is also important to consider differences between American and British English and using alternative spellings in the keywords section may be a good strategy to increase discoverability (see §1a(iv)).

A systematic approach to choosing key terms or phrases involves scrutinizing similar studies to identify the terminology predominantly used. Lexical resources or linguistic tools (e.g. Thesaurus) that provide variations of essential terms can be beneficial in this process, ensuring that a variety of relevant search terms direct readers to your work. Using tools such as Google Trends can also help identify key terms that are more frequently searched online [5].

Avoiding ambiguity also plays a crucial role in enhancing discoverability. Precise and familiar terms often outperform their broader or less recognizable counterparts. In fact, using uncommon keywords is negatively correlated with impact [35]. For example, ‘survival’ conveys a clearer meaning than the more expansive term ‘survivorship’, and ‘bird’ resonates more readily with a broader audience than the specialized term ‘avian’. Papers with these key terms are likely to appear more frequently in literature searches (but see [7] for a discussion on the consistency of search engines) and hence have the potential to be more impactful.

#### (iii) Use readable key terms

When crafting your abstract, it is crucial to prioritize the readers’ ability to discover and understand your work. Select key terms or phrases that are likely to appear in search queries, ensuring they are not separated by words or special characters that might hinder discovery [5] (figure 2). For instance, instead of ‘offspring number and survival’, consider using ‘offspring number and offspring survival’ to align with typical search queries (e.g. ‘offspring survival’). For similar reasons, avoid key terms separated by (suspended) hyphens (e.g. use ‘precopulatory and postcopulatory traits’ instead of ‘pre- and post-copulatory traits’) or containing special characters and symbols, unless they represent the most common terminology. While our minds can connect symbols and hyphenated words to their intended meaning, search engines cannot, unless these are directly specified. In fact, recent meta-analytic evidence shows that complex abstracts with low readability are penalized in terms of citation rates [23].

Technical jargon is often difficult to circumvent in the methods section but strive to minimize its use in the abstract. When technical terms or acronyms are necessary, choose them thoughtfully to avoid confusion. For example, ‘PCR’ could stand for ‘polymerase chain reaction’ or ‘principal coordinates regression’, potentially confusing a broad readership. In some instances, acronyms are a useful way to reduce word count and make the abstract readable, particularly for key terms that are mostly defined by their acronyms (e.g. per- and polyfluoroakyl substances, PFAS). In such cases, ensure that both the acronym and its definition are in the abstract and keywords, preserving clarity while maximizing discoverability (figure 2).

#### (iv) Use synonyms between sections

Leveraging synonyms is another tactic to maximize the chances of your article being discovered [5]. Readers often search for relevant studies using one or a few key terms and may miss relevant studies because these key terms are not present in the title, abstract or keywords. Therefore, including as many key terms as possible across the title, abstract and keywords will maximize the chances of a study being found [5,36]. To do this efficiently, consider all possible synonyms of key terms, using lexical resources or seeking advice from field experts and collaborators. Using text mining from samples of relevant studies is also an efficient way to find additional key terms [37].

Once your key terms are selected, strategically distribute them across your title, abstract and keywords (figure 2). Preferably, use the most common key terms in the title and abstract, as these are often ranked higher by algorithms organizing search results by relevance [5]. For readability and clarity, maintain consistent terminology in the abstract itself, but vary verbs and adjectives to keep the writing engaging. Keeping the key terminology consistent preserves the abstract’s clarity while leveraging different synonyms in other sections can enhance discoverability. In fact, evidence shows that articles that distributed key terms between sections had citation advantages [26]. On the other hand, doubling the frequency of key terms in abstracts increases citation rates by less than one percent [33]. Interestingly, article citation rates are positively (albeit weakly) related to the number of keywords [23,35], suggesting that distributing synonyms can provide advantages. This underscores the importance of careful keyword selection and placement, turning what might be overlooked as a minor detail into a meaningful opportunity to extend the impact of your work.

#### (v) Use umbrella terms carefully

As discussed previously, selecting the appropriate terminology in the title, abstract and keywords is essential to accurately represent your study and reach potential readers. This involves carefully considering the use of key terms or phrases that are directly related to your research, as well as umbrella terms that can convey broader context (figure 2). Umbrella terms are broad and general phrases that encompass a wide range of concepts. While they can be useful for situating your study in a larger framework and enhancing discoverability [35], misuse or over-reliance on these terms can render your paper vague and lead to confusion [29].

For instance, if your research specifically examines the impact of deforestation on amphibian biodiversity in a particular region, it might be appropriate to mention ‘biodiversity loss’ or ‘environmental degradation’. However, using overly broad terms such as ‘climate change’ without direct relevance could dilute the specificity of your research, distancing it from its core audience. Similarly, using broad terms, such as ‘anthropogenic impacts’, is not optimal if the study is focusing on urban ecology or eutrophication. In navigating umbrella terms, you must strike a delicate balance between providing a broader context and maintaining the specificity of your study. This will ensure that your study will be discovered by a broad audience that includes specialists and researchers from other fields.

#### (vi) Describe the key aspects of your study

A recommended approach adopted by some ecological and evolutionary biology journals is to structure the abstract using the IMRAD framework (introduction, methods, results, and discussion) or derivatives. While not all journals allow structured abstracts, any abstract can be organized logically. The IMRAD framework facilitates a logical flow of information and ensures that the abstract is a stand-alone summary of the paper. It also ensures that researchers can efficiently locate specific abstract sections and gather the necessary information (figure 2).

Within these abstract sections, key elements should be incorporated to enhance discoverability in online bibliographic databases. These include the taxonomic group, species name, response variable(s), independent variable(s), study area and study type (figure 2). By including these components, the abstract becomes more discoverable to researchers searching for a specific aspect of your study. For example, one may be interested in compiling studies on wing length in tropical birds and may search for the key terms ‘birds’, ‘wing length’ and ‘tropical’. Relevant studies using alternative key terms such as ‘passerines’ instead of ‘birds’, general terms such as ‘body size’ instead of ‘wing length’, or failing to include key terms such as ‘tropical’, may not be found. Optimally, all these keywords should be present to maximize your chances of being discovered.

However, deciding on the level of classification when describing these key sections is not trivial. For instance, taxonomic groups could be divided into formal levels of classification (order, family, genus, etc.), or more colloquially (birds, reptiles and fish). Study areas can also vary in granularity, ranging from the continent level to the local county. We recommend proofreading your abstract with a focus on discoverability. Imagine yourself looking for a similar study and consider what elements of the abstract would facilitate its discovery. You may also search for similar studies or systematic reviews and meta-analyses on related topics. Choosing the right terminology is a compromise between discoverability and specificity, and the choice of these terms should align with the scope and audience of your study.

#### (vii) State meaningful results

The presentation of results in the abstract is essential as it highlights the study’s central quantitative findings. To effectively communicate these findings, the results should be summarized concisely in one to three clear sentences, emphasizing key points and avoiding complex statistical details that might require specialized knowledge or extensive contextualization. The results should be accessible to a wide audience, especially in ecology and evolutionary biology, where readers have varying backgrounds and expertise levels.

While null hypothesis significance testing results are commonly reported in abstracts, they do not provide information on the magnitude or practical importance of the observed effect [38,39]. Instead, the focus should be on the effect size, a measure that conveys the magnitude, direction and precision of an effect. Effect sizes describe more meaningful information about the biological effect than statistical significance alone [40,41]. Therefore, if you wish to report statistical significance, *p*-values are preferable to present alongside effect sizes rather than *in lieu* of them. Unless otherwise stated, it is also often reasonably safe to assume that results presented in the abstract are statistically significant.

Consider, for example, a study on the impact of temperature changes on fish body size. Instead of reporting *p*-values or model coefficients, it would be more effective to say, ‘We found that a 3°C increase in water temperature led to a 15% (±2% SD) decrease in body length’. This statement conveys the core finding with a focus on the magnitude and direction of the effect, making it more comprehensible to a wide readership without extensive contextualization. In cases where these results are not statistically significant, one may add ‘albeit non statistically significant*’* to provide caution regarding the replicability of the results.

#### (viii) Write with clarity and a narrative

The quality of an abstract is an important factor in determining the life and legacy of a paper and requires a careful balance of accuracy, clarity and style. Though often overlooked, adding a narrative (i.e. a coherent and logical sequence of information) to your abstract can elevate its appeal. While the content must be scientifically rigorous, a well-phrased abstract can make the reading experience more engaging without sacrificing scholarly value (figure 2). In fact, narratives are inherently persuasive and favour engagement with a broader audience [11]. By weaving a clear narrative and connecting ideas, you can enhance both the readability and appeal of your work [10–12]. While narrativity is subjective, narrative indicators (i.e. metrics measuring the degree of narrativity) can be used to assess and develop your narrative and are positively correlated with journal impact factors and citation rates [10].

Importantly, the IMRAD structure often aligns with logical narrative structures. Sequentially stating elements of the introduction, methods, results and discussion can effectively weave a coherent narrative. Consider, for instance, this narrative in seven acts. First, it is important to set the scene with the general research topic or theory investigated (Introduction). Second, we may introduce the conflict, such as an important knowledge gap, or controversies in theory (Introduction). Third, we may introduce the protagonists, the species or taxonomic group used to resolve this conflict (Introduction). Fourth, we may define the quest: the general research objectives (Introduction). Fifth, we may describe the journey by detailing the methodologies employed (Methods). Sixth, we can reveal the discoveries and outline the results of the study (Results). Seventh, we can reflect on this journey, putting the results into the context of the broader literature (Discussion). In this example, using the IMRAD structure (whether the abstract is strictly structured or not) helps describe important aspects of the study, while also weaving a logical narrative.

#### (ix) Benchmark your draft

Envisioning yourself conducting a literature search can lead you to become a better craftsperson of the discoverability of your own work. A robust strategy to gauge the coverage of your key terms is to compare them with the content of similar studies. To do this, you can use your key terms in database searches (e.g. Web of Science, Scopus or Google Scholar) to inspect their effectiveness in capturing related papers on the subject you are investigating [42]. Conversely, you can use search terms of existing systematic reviews or meta-analyses relevant to your study topic to ensure that your paper will be retrieved based on your title, abstract and keywords. You can do a similar exercise by attempting to do a comprehensive search of your own to think of distinct terms you can include as keywords. You can also learn from the methods used for conducting systematic searches of literature (e.g. see [8] for a guide for ecologists and evolutionary biologists)—especially on how search terms are selected, and search strings are composed to find evidence used in meta-analyses and quantitative evidence syntheses. In addition, professional courses or advice from librarians are a great way to gather knowledge on the workings of search engines and systematic review searches. By understanding the strengths and limitations of search engines, you will increase the chances of your scientific contributions being noticed and used to inform policies, practices and scientific progress.

Remember to recognize that key terms contained in your abstract should not be duplicated as keywords. That is because the abstract content is indexed by most databases, meaning that the words in the abstract naturally act as keywords. At this stage, it is valuable to share your abstract and keywords with co-authors to seek their insights. Sharing your draft with someone outside of your field may also help find terms that are overly technical for a broad readership. This collaborative exercise is useful to ensure you use the most relevant key terms, increasing discoverability.

#### (x) Translate your content

English is considered the *lingua franca* of scientific research, allowing it to have a global reach. However, not all scientists or readers have a good understanding of English, limiting the accessibility of vital research [43]. Recognizing this, some journals allow titles and abstracts in multiple languages, although only 18% of journals in biological sciences currently offer this option [44].

Translating titles and abstracts enhances inclusivity, broadens the scope and impact of research and serves as a bridge to overcome language barriers [44–47]. The reach of scientific studies can be expanded to include scientists, practitioners, policymakers and the general public in non-English speaking regions by making content available in different languages when permitted [43–48]. This fosters a more balanced global understanding of scientific advancements. Furthermore, translating content is likely to broaden recognition, more citations and potential collaborations, amplifying the global resonance of scientific studies. Such a practice promotes equitable engagement in the scientific community, thereby increasing the visibility and impact of research. Keep in mind that the languages that are more impactful for your study’s discoverability may depend on its main topic. Particularly, we recommend translating content into the languages spoken by all co-authors, as well as languages relevant to the study area, organism or field. For instance, Spanish, Portuguese, Chinese (simplified), French and Italian are the five non-English languages most used in the conservation biology literature [47], so they would be ideal choices when translating content on this topic.

#### (xi) Further considerations

Carefully crafting your title, abstract and keywords is important for search engine optimization. However, it is not the only way to maximize the findability of scientific articles. It is also important to choose the right journal for your paper to maximize its chances of discovery. Targeting the appropriate audience, rather than merely the journal’s impact factor, should be the primary consideration. Exploring various open access options, including green open access (e.g. via preprints like bioRxiv or EcoEvoRxiv), can significantly increase the visibility and accessibility of your work [23,49,50]. Indeed, open-access publications have citation advantages over non-open-access counterparts in ecological journals [51,52]. In addition, advertising studies on social media can boost your study’s engagement. In fact, Twitter (now X) activities predict an article’s citation performance better than a journal’s impact factor [53,54]. Although citation performance does not directly relate to discoverability, discoverability is a prerequisite for being cited among the sea of scientific literature. Nevertheless, a study’s citation will ultimately be contingent upon its content; prioritizing rigorous and transparent scientific practices should always outweigh a focus on (over)optimizing discoverability.

Finally, we remind researchers to cite writing guides (including this one!) if they find them useful. Citing writing guides in the methods or acknowledgements section not only credits the authors but also enhances the discoverability and usage of such guidelines. As more people embrace these recommendations, the scientific community at large will benefit from more searchable, clear and engaging literature.

### (b) Journal policies in the ecological and evolutionary biology literature

Above, we outlined ten strategic recommendations to optimize the most prominent marketing elements of your study. Nevertheless, these strategies must navigate the limitations imposed by journal guidelines, specifically regarding constraints on the length and structure of title, abstract and keywords. To investigate these constraints, we conducted a literature survey examining the word limits of 230 journals in the fields of ecology and evolutionary biology.

#### (i) Methods

We report our methods as per the MeRIT guidelines [55]. On 2022/09/30, CW surveyed journals classified as ‘Ecology’ or ‘Evolutionary Biology’ by Clarivate Journal Citation Reports, and PPottier supplemented this list with 13 multidisciplinary journals including *Nature*, *Nature Communications*, *Nature Climate Change*, *Scientific Reports*, *Science*, *Science Advances*, *Communications Biology*, *Proceedings of the National Academy of Sciences*, *PLoS Biology*, *Biological Reviews*, *Current Biology*, *eLife* and *Philosophical Transactions of the Royal Society B—Biological Sciences*. Our aim was to compile a representative, though non-exhaustive, list of journals publishing studies in ecology and evolutionary biology.

To gauge recommended word limits, PPottier, ML, SB, SMD, ELM, ARM, KM, LR, JT, CW, YY and SN inspected the author guidelines of each journal as of 2022/11/28, quantifying the constraints on title and abstract length, and the maximum number of keywords permitted for standard research articles. Where a range was provided, we used the upper limit. In addition, we assessed whether the abstract layout was flexible or structured.

We further quantified the actual length of titles, abstracts and keywords from a sample of approximately 25 studies from each journal. PPottier conducted a range of bibliographic searches (electronic supplementary material) on 2023/09/11 in Web of Science (core collection) using The University of New South Wales’ subscription and randomly selected 25 of the most recent studies from each journal. For multidisciplinary journals, ML, SB, SMD, PAD, ELM, ARM, AM, KM, PPollo, LR, CW, YY and SN manually inspected studies indexed in Web of Science, selecting the 25 latest standard research articles in ecological and evolutionary biology published between 2022/11/28 (i.e. when journal guidelines were inspected) and 2023/09/11. From this sample of studies, PPottier identified 2321 articles with abstract lengths that differed by at least 25 words from the word limit imposed by journals. As these articles are likely to have different formatting structures than standard article types (e.g. commentaries, opinion pieces, reviews, perspectives and package descriptions), and thus adhere to different journal guidelines, they were further screened by PPottier, ML, SB, SMD, PAD, ELM, ARM, AM, KM, PPollo, LR, CW and SN. We did not screen studies from journals only publishing non-standard article types (e.g. *Trends in Ecology & Evolution* only publishes reviews and opinions) or multidisciplinary journals. We excluded 383 non-standard articles from our study sample.

Subsequently, PPottier processed the data using R statistical software [56] (version 4.3.0). PPottier used text mining to measure the length of titles, abstracts and the number of keywords using the *stringr* package [57] (version 1.5.0). Note that PPottier excluded studies with abstracts under 50 words, as they were classified as comments or opinion pieces. Furthermore, PPottier analysed whether keywords were duplicated in the title or abstract. In making comparisons between author guidelines and study samples, PPottier excluded journals without explicit word limits for titles, abstracts or keywords. PPottier also conducted a linear regression to correlate title word length with character length and employed predictions from this model (using the *predict* function) to convert character limits to word limits. In total, we obtained a sample of 5323 studies from 230 journals.

#### (ii) Journal guidelines

**Figure 3.**
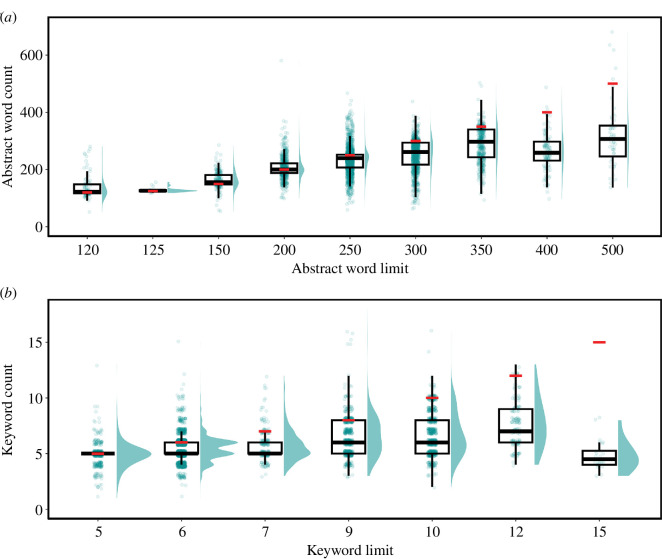
Comparison between word limits imposed by journals and the length of abstracts (*a*) and titles (*b*) in a sample of 5323 studies in ecology and evolutionary biology. Individual data points refer to abstract word or keyword counts from a sample of studies, along with their density distribution. Medians are represented by the thick black lines, interquartile ranges by the boxes, and whiskers extend to 1.5 times the interquartile range. Red lines indicate the abstract or keyword limit imposed by journals.

Journal guidelines on title, abstract and keyword limits varied greatly, with a range of 120–500 words and an average of 266.0 words (±66.9 s.d.; figure 3*a*). Most commonly (31.3%), journals adhered to an abstract limit of 250 words; and nearly a quarter (22.6%) of journals did not stipulate an abstract length limit in their guidelines. Additionally, 13.0% of journals permitted structured abstracts using the IMRAD framework or derivatives.

Titles were frequently unregulated in author guidelines, as 77.0% of journals refrained from imposing a word or character limit, at least in the author guidelines. For journals that did state word limits, titles were recommended not to exceed 15.8 words (±5.3 s.d.) on average (range: 6–33).

With regards to keywords, nearly a quarter (24.1%) of journals did not mention a limit, although it must be noted that these, as well as abstracts and keywords, may be constrained within the journal’s submission platform. In cases where journals did provide a keyword limit in their guidelines, the majority (34.8%) capped the number of keywords at six, with a range of 5–15 words and an average of 7.3 (±2.0 s.d.; figure 3*b*).

#### (iii) Study samples

Using a sample of approximately 25 recent studies from each journal, we found that the range of abstract lengths varied significantly, spanning from 52 to 1390 words and averaging 238.0 words (±79.4 s.d.; *n* = 4061; figure 3*a*). Interestingly, abstract lengths often matched restrictions set by journal guidelines; particularly for journals allowing less than 300 words (figure 3*a*). For journals with word limits equal or above 300 words, abstracts were generally shorter than the word limit (figure 3*a*). Therefore, 250 words are likely sufficient for most authors to accurately describe the content of their study.

In journals stating explicit title word limits, title lengths ranged from 2 to 33 words, with an average of 13.8 words (±4.2 s.d.; *n* = 1207). In other journals, title lengths were similar, averaging 14.8 words (±4.5 s.d.; *n* = 4116).

In our sample of studies, we found that the number of keywords averaged 5.9 (±1.6 s.d.; range 1–18; *n* = 3907; figure 3*b*). This figure is surprisingly lower than what author guidelines typically prescribe (7.3 keywords ± 2.0 s.d.; figure 3*b*). Interestingly, 92.3% of the studies in our survey (*n* = 4525) duplicated at least one keyword in either the title or abstract, which we found was occasionally recommended in journal policies. On average, 2.68 (±1.62 s.d.) keywords were reused in the title or abstract, which represents 45.8% (±0.25 s.d.) of the keywords used.

## 2. Recommendations to editors

In an era where information is increasingly digitized, publishing constraints traditionally imposed by print media may no longer be fitting. Our investigation into journal guidelines, and how authors engage with abstract length and keyword limitations, yields insights that call for a potential re-evaluation of current practices.

### (a) Adopting structured abstracts and reconsidering word limits

Our survey reveals that authors frequently push their abstracts to the maximum allowable length (figure 3*a*). This trend is especially pronounced in journals with stringent word constraints (≤200 words), indicating that current word limits may be overly restrictive. Historically, these limits were rooted in the physical space constrained by printed journals. In today’s digital landscape, such limitations are less relevant and may hinder the discoverability and citation of research.

We encourage editors to consider adopting (optional) structured abstracts, which often have the advantage of ensuring that authors do not omit to specify key aspects of their study [58,59]. In the field of ecology and evolutionary biology, information such as the taxonomic group, species name, location, study type and variables investigated are essential study aspects that should always be stated. Given that structured abstracts are typically longer [60] and that authors already approach word limits, editors may need to consider relaxing word count constraints. As demonstrated with examples using abstract lengths of 120 and 250 words (figure 2), an increase in word count can allow authors to supplement their abstract with additional key terms. This adjustment could significantly enhance the discoverability of studies not only for regular author searches but also for systematic reviews. Similar to title characteristics, evidence linking abstract length to impact metrics is mixed [23,61–63]. However, longer abstracts generally tend to be positively related to citation rates, although the effects are only weak or moderate [23,63,64]. This suggests that the content of the abstract is likely more important than its length. Consequently, offering the opportunity for authors to describe their content more accurately may facilitate discovery and impact.

### (b) Optimizing keyword usage

Most journals limit the number of keywords, constraining the study’s association with synonyms and relevant terms. We recommend that journals implement a large keyword limit to enhance discoverability. In fact, recent evidence suggests that the number of keywords is positively correlated with citation rates [23,35,64]. Increasing keyword limits is also perhaps easier to implement than increased abstract lengths. In fact, we believe there are no clear incentives to restrict the number of keywords, and both authors and journals could benefit from increased discoverability. Implementing a standardized term system that is machine-readable, akin to MeSH terms for biology, could also help authors choose the right terminology and increase indexing. Our survey also revealed that authors generally do not use all the keywords allowed. We argue that this is a missed opportunity. We encourage authors, editors and reviewers to leverage the potential of strategic and comprehensive keyword selection.

A concerted effort by editors and reviewers to assess keywords (as well as key terms in the abstract and title) for relevance, accuracy and redundancy can further ensure that these terms genuinely reflect the study’s content and optimize discoverability. In fact, 92% of the studies we surveyed used redundant key terms between the title, abstract and keyword sections, although this can negatively influence discoverability and impact [26]. Ensuring that the right keywords are used and placed thoughtfully in these critical places of a study would be an important step to increase the discoverability and impact of scientific publications.

### (c) Accepting multilingual content

Publishing multilingual abstracts and titles could significantly amplify the global resonance of scientific studies, enhancing accessibility across linguistic barriers. However, only 18% of journals in biological sciences allow multilingual abstracts [44]. We encourage editors to consider publishing multilingual summaries to increase the accessibility of scientific knowledge in countries where English is not the primary language, potentially yielding a greater impact [44–48]. For instance, FEMS (Federation of European Microbiological Societies) journals have translated the abstracts and titles of numerous articles in Portuguese and Spanish, which has significantly increased knowledge discovery (https://academic.oup.com/fems-journals/pages/alam_2018; accessed on 2023/08/23). As translation tools have yet to properly incorporate highly specific and complex terminology in scientific research, we believe it is valuable to allow authors to submit multilingual content. By embracing multilingual abstracts and titles, editors can foster greater inclusivity and bridge the language divide, enriching the global scientific dialogue and allowing valuable research to reach an even wider audience.

## 3. Conclusions

Crafting a title, abstract and choosing the right keywords is an art in itself. By understanding how scientific studies are indexed in databases and searched by authors, we can strategically increase the discoverability and impact of scientific research. Particularly, the strategic use and placement of keywords can maximize indexing, in turn laying the groundwork for discoverability and impact. Comparing author guidelines with samples of studies from journals in ecology and evolutionary biology, we found that authors often push their abstract to the maximum word limit allowed and that the number of keywords used is low and mostly redundant. These reflect restrictive guidelines that may be relics of the print era when physical page limits existed and are not optimized to increase the discoverability of studies in the rapidly expanding landscape of digital publications. Therefore, we encourage journals to use effective strategies to maximize the impact of their publications. By embracing these recommendations, editors can create an environment that aligns with the digital era and promotes the broader dissemination and impact of scientific research. Such actions reflect a recognition of the evolving needs of the scientific community and the critical role of discoverability in shaping the scientific knowledge landscape.

## Data Availability

Data, code and additional materials are available [65] and are archived permanently in Zenodo [66]. Supplementary material is available online [67].
